# The oxidative status and inflammatory level of the peripheral blood of rabbits infested with *Psoroptes cuniculi*

**DOI:** 10.1186/1756-3305-7-124

**Published:** 2014-03-25

**Authors:** Xiaofei Shang, Dongsheng Wang, Xiaolou Miao, Xuezhi Wang, Jianxi Li, Zhiqiang Yang, Hu Pan

**Affiliations:** 1 Key Lab of New Animal Drug Project, Lanzhou 730050, Gansu Province, People's Republic of China; 2 Key Laboratory of Veterinary Pharmaceutical Development, Ministry of Agriculture, Lanzhou Institute of Husbandry and Pharmaceutical Sciences of Chinese Academy of Agricultural Science, Lanzhou 730050, People's Republic of China

**Keywords:** Rabbits, *Psoroptes cuniculi*, Oxidative status, Superoxide dismutase, Inflammatory factor

## Abstract

**Background:**

*Psoroptes cuniculi* can parasitise the ear canal of the rabbit, and cause the afflicted animals to cease feeding and become severely debilitated, sometimes resulting in death. In this study, we examined the oxidative status and inflammatory level of the peripheral blood of rabbits infested with *Psoroptes cuniculi* and investigated the pathogenesis of this disease.

**Methods:**

A total of 24 rabbits were divided into a healthy rabbit group and two infested rabbit groups. After weighing the rabbits, approximately 5 ml of blood was obtained from each animal. Then, the blood serum was extracted and used to assess the levels of antioxidant enzymes and inflammatory factors.

**Results:**

Compared to the healthy rabbits, the activities of catalase and glutathione-S-transferase and the level of malonyldialdehyde were increased, but the activity of superoxide dismutase was reduced in the infested rabbits. At the same time, a variety of inflammatory cells were activated, and the levels of inflammatory factors such as prostaglandin E_2_, interleukin-6, interleukin-8 and transforming growth factor-β1 were increased in peripheral blood.

**Conclusion:**

Animal acariasis was associated with immunosuppressive disorders and inflammatory reaction. These results advance our understanding of the pathogenesis of *Psoroptes cuniculi* infestation in rabbits and can help guide the effectual treatment of this disease in clinics.

## Background

**Figure 1:**
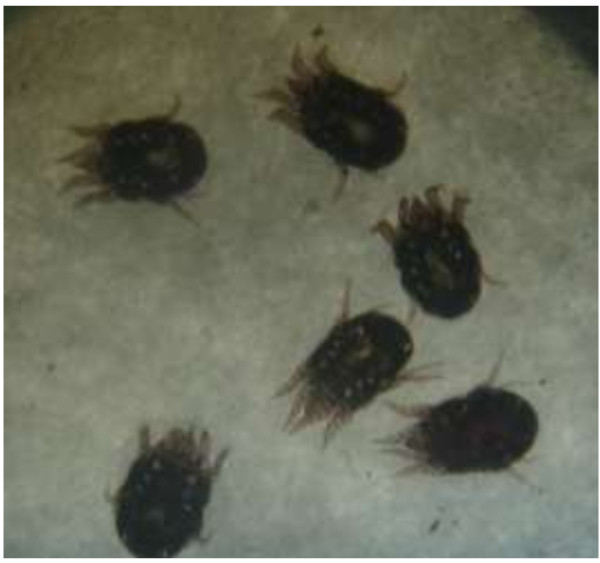
**The picture of *Psoroptes cuniculi *** under microscope (16*10).

Animal acariasis is a veterinary skin disease that can reduce the productivity and the quality of animal products [1]. As a pathogen, *Psoroptes* parasitizes the body surface or the epidermis of sheep, horse, rabbit, goat, cattle and buffalo, etc., causing the afflicted animals to cease feeding and become severely debilitated. In rabbits, *Psoroptes cuniculi* can damage the pineal layer of the rabbit ear. Infestation can occur by direct contact with infested rabbits or by contact with infected bedding, especially for young rabbits and peaked rabbits (Figure 1). If the infestation is not treated, it may cause serious economic loss due to decreased food consumption and the development of meningitis or the death of the animal [2]. It is therefore necessary to study the pathogenesis of this disease for developing the new drug or the therapeutic method.

Now, more and more scientific reports have focused attention on sarcoptic mange in animals, it is thought that the disease is associated with immunosuppressive disorders [3]. In addition, the immune status of the animal, the nutritional status and oxidative stress may play very important roles in the pathogenesis of this disease [4-7]. However, up to now, except for a study by Singh *et al*. that proposed that a significant alteration of the oxidant/antioxidant balance is a factor in the pathogenesis of *P. cuniculi* infestation of rabbits and that recovery could be enhanced by combining ivermectin treatment with vitamin A, D_3_, E, and H supplementation [8], no other studies have examined the oxidative status or levels of inflammatory factors in the peripheral blood of rabbits infested with *P. cuniculi* to our knowledge.

As we know, once animals are infested with *Psoroptes cuniculi,* they would and would induce an immune reaction by mites. Then, the oxidant/antioxidant balance in animals would be disturbed and some oxidative substances would be constantly generated *in vivo*, such as reactive oxygen species (ROS) [8,9]. When these substances generated overloaded the antioxidant defense, the free radicals could interact with endogenous macromolecules and alter the cellular functions, and induce some serious adverse effects on the skin, including edema, erythema, wrinkling, inflammation, autoimmune reaction, etc. [10,11]. At the same time, animals would develop the anti-oxidative mechanisms to minimize oxidative damage, some enzymes and anti-oxidant factors would be activated or released, such as glutathione peroxidase (GSH-Px), glutathione-S-transferase (GST), superoxide dismutase (SOD) [12]. However, how the oxidative status and inflammation levels change, and which anti-oxidant and inflammatory factors are activated or released are still unclear.

In this paper, we studied the oxidative status and inflammation levels of rabbits with psoroptic mange by determining the activities of the antioxidant enzymes and the levels of inflammatory factors in the peripheral blood. Our goal was to develop a better understanding of the pathogenesis of this disease.

## Methods

### Experimental animals and groups

Rabbits (80–100 days of age) were obtained from the Experimental Animal Center, Lanzhou Institute of Biologicals (Lanzhou, China). The experiments complied with the rulings of the Gansu Experimental Animal Center (Gansu, China) and were officially approved by the Ministry of Health, P.R. China in accordance with NIH guidelines.

In this study, all of the diseased rabbits were divided into two groups (scores of 3 and 6). Each group consisted of 8 rabbits, and 8 healthy rabbits were used as the healthy control group. They were kept in cages where they had free access to food and water, and they were maintained on a 12 h light/dark cycle.

### Selected rabbits

The inclusion criteria for the animals selected for this study have been previously described by Fichi *et al.* and Shang *et al*. [13,14]. Briefly, before enrolment in the study, all of the rabbits were examined by clinical and dermatological methods with an otoscope and a microscope. The rabbits were naturally infested with *Psoroptes cuniculi* and suffered from the clinical disease for at least 10 days before presentation. None of the rabbits had been treated with ectoparasiticides or steroidal anti-inflammatory drugs in the 30 days before the blood samples were drawn. The degree of infestation was evaluated according to a previously described scoring system: 0 indicated an absence of scabs and or mites; 0.5 was irritation in the ear canal but no mites observed; 1 was a small number of scabs in the ear canal, mites present; 2 was external ear canal filled with scabs, mites present; 3 was scabs in ear canal and proximal 1/4 of the pinna, mites present; 4 was pinna half-filled with scabs, mites present; 5 was 3/4 of the pinna filled with scabs, mites present; 6 was the entire internal surface of the pinna covered with scabs, mites present [9]. In this study, samples were collected from diseased animals with scores of 3 and 6 and from healthy animals with scores of 0 that were free of other diseases, as determined by laboratory and clinical examination. After the samples were collected, the rabbits were treated immediately.

### Weight and blood samples

After careful laboratory and clinical examination, all of the rabbits were weighed. Then, 3% pentobarbital sodium (30 mg/kg) was used for the sedation of rabbits and approximately 5 ml of blood was obtained from the auricular vein of each animal using a sterile needle into tubes. After standing for 30 min, the serum was extracted from the blood samples and stored at −20°C for up to 5 days. It was used to assay the oxidative stress and inflammatory factors promptly.

### Assay for oxidative stress

After collecting the blood serum, 100 μl aliquots were used to determine the activities of superoxide dismutase (SOD), catalase (CAT), glutathione-S-transferase (GST) and malonyldialdehyde (MDA) with a Nanjing Jiancheng assay kit (Nanjing Jiancheng Bioengineering Institute, Jiangsu, China). Detailed, SOD activity in blood serum was measured by using nitro blue tetrazolium as a substrate after suitable dilution with SOD assay kit (Lot. 20130424), the increase in absorbance was scanned with an ultraviolet spectrophotometer (Evolution 300 UV–VIS, Thermo Scientific, U.S.A.) at 550 nm. One unit of SOD activity was defined as the amount of enzyme that inhibited autooxidation by 50% under the given experimental condition and the values were expressed as U/ml. CAT activity in blood serum was estimated by using H_2_O_2_ as a substrate with CAT assay kit (Lot. 20130506), and the absorbance was scanned at 405 nm by ultraviolet spectrophotometer. GST activity was determined by assaying the concentration of GSH with GST assay kit (Lot. 20130528), and the absorbance was scanned at 412 nm by ultraviolet spectrophotometer. The concentration of MDA, a reliable marker of lipid peroxidation, was estimated in blood serum following the manual of Nanjing Jiancheng MDA assay kit (Lot. 20130407). Optical density was measured using an ultraviolet spectrophotometer at 532 nm against blanks prepared by using distilled water. The activities of SOD, CAT and GST and the level of MDA were calculated from the resulting absorbance values.

### Assay for inflammatory factors

After collecting the blood serum, 10 μl aliquots were used to determine the levels of prostaglandin E_2_ (PGE_2_), interleukin-6 (IL-6), interleukin-8 (IL-8) and transforming growth factor-β1(TGF-β1) with rabbit ELISA PGE_2_ assay kit, rabbit ELISA IL-6 assay kits, rabbit ELISA IL-8 assay kits and rabbit ELISA TGF-β1 assay kits according to the manufacturer’s instructions (R&D System, U.S.A.), respectively. Briefly: firstly 10 μl sample serum with 40 μl sample diluents were added to 96-well plates (50 μl standard solutions). Following incubation for 30 min at 37°C, the solutions were removed and the plate was washed 5 times with wash solution. 50 μl HRP-conjugate reagents were then added to each well, and after incubating for 30 min for 37°C and washing 5 times again 50 μl chromogen solutions A and B were added and incubated for 10 min at 37°C in the dark. Finally, 50 μl of stop solution was added to each well, and the optical density was measured at 450 nm within 15 min using a microplate reader Multiskan MK3 (Thermo Scientific, U.S.A.), and the levels of PGE_2_, IL-6, IL-8 and TGF-β1 were calculated from the resulting absorbance values.

### Statistical analyses

The data obtained were analyzed using SPSS software version 13.0 and expressed as the mean ± SD. The data were analyzed by a one-way ANOVA followed by Student’s two-tailed *t*-test for the comparison between test and control, and Dunnett’s test was used when the data involved three or more groups. P-values of less than 0.05 (*P < 0.05*) were considered significant.

## Results

### Weight of the rabbits

We weighed all of the rabbits included in this study, which were selected for 80–100 days of age. The results showed that the healthy rabbits had an average weight of 2.15 ± 0.410 kg, and the weights of the rabbits infested with *P. cuniculi* with clinical scores of 6 and 3 were significantly decreased to 1.35 ± 0.404 kg (*P < 0.01*) and 1.64 ± 0.302 kg (*P < 0.05*), respectively. This reduction in weight may be related to the oxidative stress and inflammation induced by the mites and if the degree of disease was more serious, the weight of rabbits was lighter.

### Assay for oxidative stress

We determined the activities of three antioxidant enzymes. The results demonstrated that as an important metalloenzyme, the activity of SOD in the infested rabbits with clinical scores of 3 (1.868 ± 0.073 U/ml) and 6 (1.831 ± 0.087 U/ml) was weakly inhibited but not significantly different from that observed in the healthy rabbits (1.918 ± 0.061 U/ml). However, the GST activity was significantly elevated in the infested rabbits with scores of 3 (79.938 ± 3.256 U/ml) and 6 (125.138 ± 4.094 U/ml) compared to the healthy controls (67.783 ± 3.694 U/ml) (*P < 0.01*). Meanwhile, the CAT activity in the infested rabbits with scores of 6 (26.258 ± 1.175 U/ml) was increased compared to the healthy controls (21.335 ± 1.135 U/ml) (*P < 0.01*). This was most likely due to the increased biosynthesis of the enzyme in response to H_2_O_2_ and other free radicals associated with *P. cuniculi* infestations. The MDA levels were assessed to evaluate the degree of lipid peroxidation in the body and to determine the potential role of lipid peroxidation in the pathogenesis of the infested rabbits. The result showed that the level of MDA in the infested rabbit with scores of 3 (4.675 ± 0.244 nm/ml) and 6 (5.195 ± 0.396 nm/ml) was markedly increased compared to the healthy rabbits (2.835 ± 0.180 nm/ml) (*P < 0.01*) (Table 1).

**Table 1:** The activities of antioxidant enzymes (superoxide dismutase, catalase and glutathione-S-transferase) and the levels of malonyldialdehyde in the peripheral blood of infested rabbits and healthy rabbits

**Oxidative factor**	**Group A**	**Group B**	**Group C**
	**(Healthy rabbits)**	**(Infested rabbits with 3 score)**	**(Infested rabbits with 6 score)**
SOD (U/ml)	1.918 ± 0.061	1.868 ± 0.073	1.831 ± 0.087
CAT (U/ml)	21.335 ± 1.135	21.284 ± 1.115	26.258 ± 1.175^**^
GST (U/ml)	67.783 ± 3.694	79.938 ± 3.256^**^	125.138 ± 4.094^**^
MDA (nmol/ml)	2.835 ± 0.180	4.675 ± 0.244^**^	5.195 ± 0.396^**^

^**^P < 0.01 compared with control.

### Assay for inflammatory factors

Finally, we determined the level of four inflammatory factors in the peripheral blood of rabbits. The results demonstrated that compared to the healthy rabbits (0.0880 ± 0.0072 mg/L, 0.0198 ± 0.0068 mg/L), the levels of the inflammatory factors IL-6 and TGF-β1 were markedly increased in the peripheral blood of the infested rabbits with scores of 3 (0.1564 ± 0.0074 mg/L, 0.0450 ± 0.0061 mg/L) and 6 (0.2274 ± 0.0118 mg/L, 0.1048 ± 0.0341 mg/L) (*P < 0.01*). The levels of PGE_2_ and IL-8 were only significantly elevated in the infested rabbits with a score of 6 (0.4612 ± 0.0128 mg/L, 0.0554 ± 0.0069 mg/L) compared to the healthy controls (0.2670 ± 0.0131 mg/L and 0.0330 ± 0.0083 mg/L) (*P < 0.01*) (Table 2).

**Table 2:** **The levels of inflammatory factors (prostaglandin E**_**2**_**, interleukin-6, interleukin-8 and transforming growth factor-β1) in the peripheral blood of infested rabbits and healthy rabbits**

**Inflammatory factor**	**Group A**	**Group B**	**Group C**
	**(Healthy rabbits)**	**(Infested rabbits with 3 score)**	**(Infested rabbits with 6 score)**
PGE_2_ (mg/L)	0.2670 ± 0.0131	0.3000 ± 0.0120	0.4612 ± 0.0128^**^
IL-6 (mg/L)	0.0880 ± 0.0072	0.1564 ± 0.0074^**^	0.2274 ± 0.0118^**^
IL-8 (mg/L)	0.0330 ± 0.0083	0.0438 ± 0.0056	0.0554 ± 0.0069^**^
TGF-β1(mg/L)	0.0198 ± 0.0068	0.0450 ± 0.0061^**^	0.1048 ± 0.0341^**^

^**^P < 0.01 compared with control.

## Discussion

As a chronic skin disease, animal acariasis is induced by mites of Sarcoptidae and Psoroptidae when they were parasites in the surface or epidermis of animals. It can lead to acute pruritus and dermatitis of hosts [15]. Although rabbit ear mange begins as a topical disease, it can induce an inflammatory response and break the oxidant/antioxidant balance of animals as the illness progresses, even leading to systemic disease.

It is known that inflammatory cells are activated as a result of inflammation in animals with mange, and recruit neutrophils and macrophages with reactive oxidants, such as hydrogen peroxide (H_2_O_2_), hypochlorite, and oxygen radicals. These reactive oxygen substances produced by cells of the immune system show potent cytotoxic effects on parasites and other pathogenic organisms [16]. Meanwhile, animals would develop the anti-oxidative systems to minimize oxidative damage by activating some enzymes and releasing anti-oxidant factors. In our study, when rabbits were infested with mites, the oxidative and anti-oxidative mechanisms were activated. The activities of CAT and GST in the peripheral blood were increased, and the activity of SOD was reduced compared to the healthy rabbits. Generally, changes in the activities of these antioxidant enzymes could break the oxidant/antioxidant balance in infested rabbits and exhaust the antioxidant system. Thus, this balance would shift towards oxidative stress. At the same time, as an indicator of oxidative stress in cells and tissue, the levels of MDA in the infested rabbits was markedly increased, and this is associated with the deterioration of cells, the development of skin lesions and the clinical manifestation of mange [17]. These results demonstrated that infestation stimulates oxidative stress, changes the antioxidant system and increases the level of lipid peroxidation throughout the body [16].

When the oxidant/antioxidant balance of animals is disrupted, overproductions of free radicals by the inflammatory cells are recruited to combat the parasites and consequent exhaustion of the antioxidant system of the infested rabbits. Meanwhile, a variety of inflammatory cells are activated, and this induces or activates various oxidant-generating enzymes to kill intra-cellular and extra-cellular parasites [18]. Subsequently, the inflammatory responses of the body are activated. In this process, components of the pro-inflammatory response to pathogens have been identified within circulating blood cells in hosts and these have been classified as the ‘systemic inflammatory response’, and some circulating leukocyte populations would present in the blood [19,20]. Meanwhile, the activity of circulating leukocytes contributes to the levels of cytokines and other pro-inflammatory markers, both systemically and at local sites of inflammation. As an important cell growth and regulatory factor, PGE_2_ plays a role in immunosuppression and anti-inflammatory effects. In our study, the levels of PGE_2_ of the infested rabbits were increased compared to the healthy rabbits. Meanwhile, as the important cytokines we observed that the levels of IL-6, IL-8 and TGF-β1 were increased compared to those in the healthy rabbits. These cytokines are released in response to the deterioration of cells and the development of skin lesions, together with the host-parasite interaction and immune-compromisation. Subsequently, the release of inflammatory factors or cytokines further activates the immune response, and excacerbates the disease. At the same time, the weight of the infested rabbits decreases as the animals became anorexic. If the infestation is not treated, it may cause serious loss, even death.

Now, more and more people are aware that animal acariasis induced by mites is not just the topical disease; but can also lead to some systemic disease, especially the oxidative response. In 2008 and 2012, Singh *et al*. and Kanbur *et al*. reported that after administrating vitamins as an adjunctive remedial to rabbits, the anti-oxidant activity and the recovery of the disease would be enhanced [8,17]. Then, Burgess *et al.*[21] thought that *Psoroptes ovis* would result in the host (sheep) systemic inflammatory response. In our study, the results indicated that psoroptic mange was associated with immunosuppressive disorders and inflammatory reaction, and the oxidant/antioxidant balance and the inflammatory factors of the rabbits infested with *P. cuniculi* were disrupted and released in the development of this disease, respectively. Based on the results, the administration of antioxidants, anti-inflammatory drugs or vitamins in conjunction with acaricides may improve the outcome of the disease. Similarly, treatment that is initiated at the early stage of the disease results in a better outcome.

## Conclusion

In summary, the rabbits infested with *P. cuniculi* lost a dramatic amount of weight, demonstrated oxidative stress and released inflammatory factors into the peripheral blood. This caused extensive cellular damage throughout the body and resulted in compromised immune and inflammatory reactions. These processes may contribute to the pathogenesis of psoroptic mange.

## Competing interests

All authors declare that they have no competing interests.

## Authors’ contributions

XS and DW conceived the study, XW and XM determined the index, XS, JL and ZY wrote the manuscript, HP performed statistical analyses. All the authors read and approved the final version of the manuscript.
